# Down-regulation of nuclear HMGB1 reduces ischemia-induced HMGB1 translocation and release and protects against liver ischemia-reperfusion injury

**DOI:** 10.1038/srep46272

**Published:** 2017-04-06

**Authors:** Guangyuan Zhao, Cheng Fu, Lu Wang, Lan Zhu, Yutao Yan, Ying Xiang, Fang Zheng, Feili Gong, Song Chen, Gang Chen

**Affiliations:** 1 Institute of Organ Transplantation, Tongji Hospital, Tongji Medical College, Huazhong University of Science and Technology, Wuhan, China; 2 Key Laboratory of Organ Transplantation, Ministry of Education, China; 3 Key Laboratory of Organ Transplantation, Ministry of Health, China; 4 Department of Immunology, Tongji Medical College, Huazhong University of Science and Technology, Wuhan, China

## Abstract

Hepatocyte-specific HMGB1 deletion has been found to worsen the injury and inflammation in liver ischemia-reperfusion injury (IRI), highlighting a role for intracellular HMGB1 in cellular protection. Down-regulation of nuclear HMGB1 by small interfering RNA (siRNA) might not only decrease its injurious extracellular role by reducing its release but also serve to maintain its beneficial intracellular role, thus protecting against IRI. We established a non-lethal liver IRI model in mice via segmental hepatic warm ischemia for 1 h and reperfusion for 6 h. HMGB1-siRNA achieved a reduction of ~60–70% in the nuclear HMGB1 expression in the liver at 48 h post-treatment. Knockdown of nuclear HMGB1 expression dramatically reduced both the degree of nuclear-cytoplasmic translocation of HMGB1 during hepatic ischemia and of HMGB1 release after hepatic reperfusion, resulting in significant preservation of liver function and a marked reduction in pathological damage. Also, HMGB1-siRNA pretreatment markedly inhibited the increases in hepatic expression of TLR4, TLR2, RAGE, TNF-α, IL-1β, IL-6, MCP-1, iNOS, and COX-2 seen in control mice after hepatic reperfusion. We demonstrated for the first time that down-regulation of nuclear HMGB1 reduces ischemia-induced HMGB1 release and protects against liver IRI, which is helpful for better understanding the role of HMGB1 in organ IRI.

As an inevitable process that occurs during liver transplantation, ischemia-reperfusion injury (IRI) represents the main cause of graft dysfunction post-transplantation^1^. The destructive effects of liver ischemia-reperfusion (I/R) mainly include initial direct cellular damage caused by ischemia and later hepatic injury resulting from inflammatory responses following reperfusion^2^,^3^.

High-mobility group box 1 (HMGB1) is an abundant non-histone nuclear protein that is mainly expressed in the nuclei of eukaryotic cells and plays an important role in the regulation of transcription^4^. HMGB1 can be either actively or passively released into the extracellular milieu, where it acts as an essential damage-associated molecular pattern (DAMP) molecule that can activate proinflammatory signaling pathways by interacting with certain pattern recognition receptors, such as Toll-like receptor 4 (TLR4), Toll-like receptor 2 (TLR2), and the receptor for advanced glycation end-products (RAGE)^5^,^6^,^7^,^8^,^9^,^10^. In recent years, evidence has accumulated in support of the notion that HMGB1 is an early critical mediator of injury and inflammation following I/R of the liver, kidney, brain, and heart^11^,^12^,^13^,^14^,^15^,^16^. In a mouse model of liver IRI, an increased level of HMGB1 was found to be released in the early period after reperfusion, and administration of neutralizing antibodies against extracellular HMGB1 provided significant protection against liver damage after I/R^11^. Thus, targeting HMGB1 may represent an effective strategy to minimize organ damage during liver transplantation.

In addition to the blockade of extracellular HMGB1 using neutralizing antibodies, an alternative approach is to prevent the release of nuclear HMGB1. We have shown previously that carbon monoxide can protect against lethal renal IRI^17^. This remarkable protective effect is associated with significant prevention of nuclear-cytoplasmic translocation and release of HMGB1, indicating that a therapeutic tool capable of inhibiting its nuclear-cytoplasmic translocation and release from ischemic cells may have a potent and efficient protective effect on organ IRI. Thus, deleting or reducing nuclear HMGB1 is a more direct way to prevent HMGB1 nuclear-cytoplasmic translocation and release.

For the purposes of research into HMGB1 function, using HMGB1 knockout mice is an ideal way to fully block the release of HMGB1. However, such knockout mice are not available because they die shortly after birth as a result of lethal hypoglycemia^18^. As an alternative, novel hepatocyte-specific HMGB1 knockout mice have been developed to investigate the role of HMGB1 within hepatocytes subjected to I/R. Surprisingly, hepatocyte-specific HMGB1 deletion actually worsens the injury and inflammation in liver I/R, resulting at least in part from increased DNA damage and nuclear instability, with a resulting increase in histone release^19^; the results of this study have suggested that HMGB1 might serve two very different roles after a sterile inflammatory insult, a beneficial intracellular role and an injurious extracellular role.

Small interfering RNA (siRNA) is a widely used tool to down-regulate a target gene in order to produce the effect of gene silencing. Although siRNA can suppress the expression of its target gene to a remarkable extent, the inhibition is not complete^20^,^21^,^22^. Therefore, we have hypothesized that down-regulation of HMGB1 by specific siRNA might not only decrease its injurious extracellular role by reducing its nuclear-cytoplasmic translocation and release but also maintain its beneficial intracellular role, thereby providing significant protective effect against hepatic IRI. In the present study, we tested this hypothesis in mice using HMGB1-specific siRNA in a hepatic warm IRI model.

## Results

### Silencing HMGB1 in vivo using siRNA

**Figure 1:**
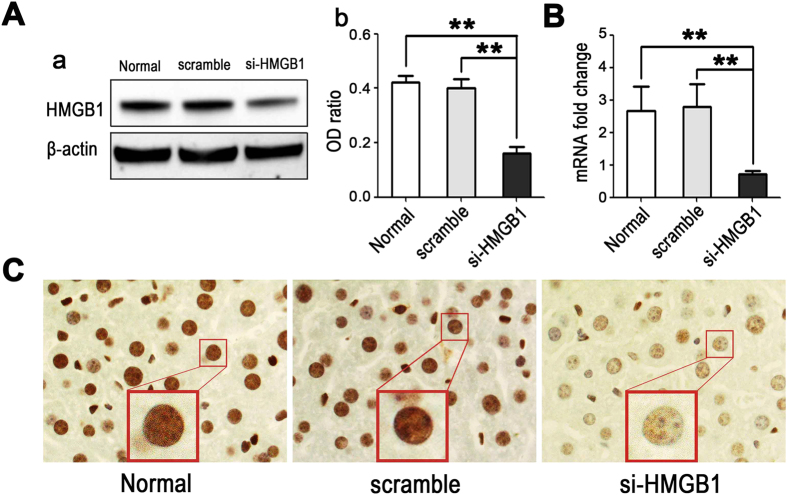
*In vivo* knockdown of nuclear HMGB1 expression in hepatocytes using siRNA. Mice were injected intravenously with 50 μg of HMBG1-siRNA (si-HMGB1) or scrambled siRNA (Scramble), and liver tissues were harvested 48 h later. Liver tissues from healthy mice who received no treatment served as controls (Normal). (**A**) Western blot analysis for HMGB1 was performed using the total cellular protein in the liver tissues, with each lane representing a separate animal. The blots shown are representative of three experiments with similar results (a). The corresponding densitometric analyses are shown as bar graphs (b). The means ± SEM are reported (**P < 0.01, n = 3 per group). (**B**) Real-time PCR was performed to measure mRNA expression of HMGB1 (**P < 0.01, n = 6 per group). (**C**) *In situ* immunohistochemical staining showed much weaker positive staining of HMGB1 in the nucleus of hepatocytes in HMGB1-siRNA-treated mice than in normal untreated or scrambled siRNA- treated mice (magnification, x1000).

To investigate whether our designed HMGB1-siRNA could effectively silence hepatic HMGB1 expression *in vivo*, we treated mice (n = 6) with 50 μg of HMGB1-siRNA or scrambled siRNA and harvested liver tissues 48 h later. The silencing efficacy was evaluated by western blotting, real-time PCR, and immunohistochemical staining. Both western blotting and real-time PCR showed that the expression of HMGB1 in the livers was significantly down-regulated (by ~60–70%) after treatment with HMGB1-siRNA (*p* < 0.01, vs. the normal untreated control group or scrambled siRNA group, Fig. 1A,B). These results were further confirmed by *in situ* immunohistochemical staining, which showed much a weaker positive staining for HMGB1 in the nuclei of hepatic parenchymal cells in HMGB1-siRNA-treated mice than those of in normal untreated or scrambled siRNA-treated mice (Fig. 1C).

### Down-regulation of HMGB1 protects against hepatic IRI

**Figure 2:**
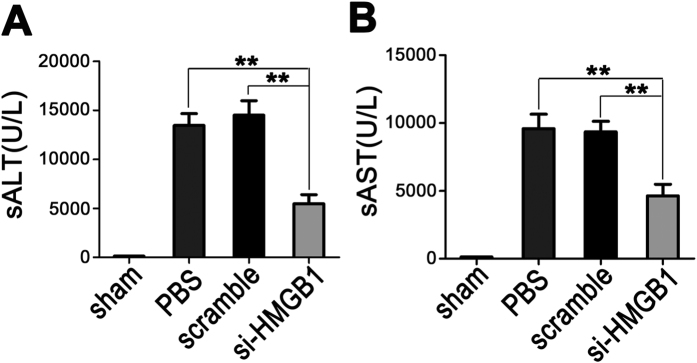
Down-regulation of nuclear HMGB1 protects against hepatic IRI. After 60 min of hepatic warm ischemia and reperfusion, the serum ALT (**A**) and AST (**B**) levels of sham-operated (sham), PBS-treated (PBS), scrambled siRNA-treated (scramble), and HMGB1 siRNA-treated (si-HMGB1) mice at 6 h after reperfusion were measured to assess the degree of liver dysfunction (**P < 0.01, n = 9 per group).

To determine whether gene silencing of HMGB1 could protect against liver IRI, mice were given PBS, scrambled siRNAs, or HMGB1-siRNA 48 h before ischemia, and serum ALT and AST levels were measured to assess the degree of liver dysfunction in each group. As compared to sham-operated mice, both PBS- and scrambled siRNA-treated mice exposed to 60 min of warm hepatic ischemia, followed by 6 h of reperfusion, showed markedly increased serum alanine aminotransferase (sALT) and serum aspartate transaminase (sAST) levels. In contrast, pretreatment with HMGB1-siRNA resulted in significant protection from hepatic injury, as evidenced by much lower levels of both serum ALT and AST in this group than in either the PBS- or scrambled siRNA-treated groups (*p* < 0.01) (Fig. 2).

### Down-regulation of HMGB1 attenuates I/R-induced pathologic damage

**Figure 3:**
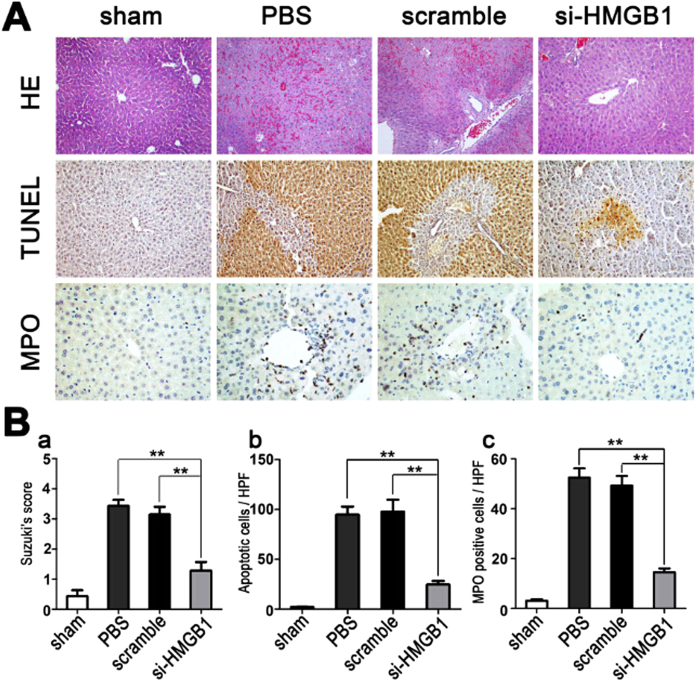
Down-regulation of HMGB1 attenuates I/R-induced pathologic damage. Mouse livers were subjected to 60 min of ischemia, followed by 6 h of reperfusion. The livers were harvested from the unclamped mice (sham), or mice clamped and pretreated with PBS, scrambled siRNA (scramble), or HMGB1-siRNA (si-HMGB1). (**A**) Hematoxylin and eosin (H&E) staining was performed to evaluate the pathological changes associated with liver damage (original magnification, x200); *in situ* TUNEL assays were performed to assess hepatic cell apoptosis (original magnification, x400); MPO assays were performed to evaluate liver neutrophil infiltration (original magnification, x400). Images are representative liver sections from 9 mice per group. (**B**) Quantitative assessment of liver necrosis (a), apoptotic cells (b), and MPO-positive cells (c), performed as described in Methods (**P < *0.01*, n = 9 per group). The data shown are means ± SEM.

Hematoxylin and eosin (H&E) staining was performed to assess the pathological changes associated with hepatic IRI. Both PBS- and scrambled siRNA-treated control mice displayed severe sinusoidal congestion and hepatocellular necrosis at 6 h after liver I/R, which was markedly attenuated in HMGB1 siRNA-pretreated mice (Fig. 3).

We also performed myeloperoxidase (MPO) assays and *in situ* TUNEL assays to assess neutrophil infiltration and hepatocyte apoptosis, respectively. HMGB1-siRNA pretreatment dramatically suppressed neutrophil infiltration and hepatic cell apoptosis, as compared to treatment with scrambled siRNAs or PBS, after 6 h of reperfusion (Fig. 3).

### Knockdown of nuclear HMGB1 expression reduces the degree of nuclear-cytoplasmic translocation of HMGB1 during hepatic ischemia

**Figure 4:**
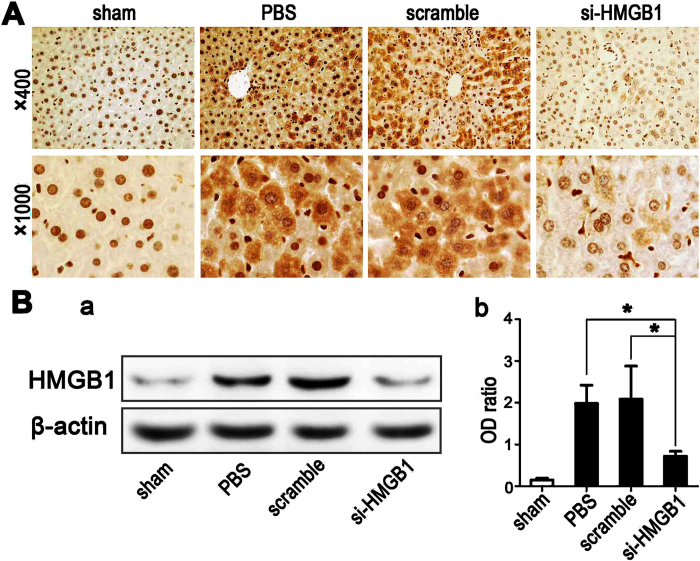
Knockdown of nuclear HMGB1 expression reduces the degree of HMGB1 nucleocytoplasmic shuttling during hepatic ischemia. Mouse livers were subjected to 60 min of ischemia without reperfusion. (**A**) Immunohistochemical staining of HMGB1 from sections of livers harvested from the unclamped sham-operated mice (sham) or from mice clamped and pretreated with PBS, scrambled siRNA (scramble), or HMGB1-siRNA (si-HMGB1) (original magnification, x400 and x1000). Images are representative liver sections from 9 mice per group. (**B**) Western blot analysis for HMGB1 was performed using cytoplasmic protein samples from sham-operated livers or the ischemic livers from PBS-, scrambled siRNA- or HMGB1-siRNA-pretreated mice, with each lane representing a separate animal (a). The blots shown are representative of three experiments with similar results. The corresponding densitometric analyses are shown as bar graphs (b). Means ± SEM are reported (*P < *0.05*, n = 3 per group).

To determine whether the gene silencing of HMGB1 could reduce the degree of early nuclear-cytoplasmic trafficking of HMGB1 in clamped ischemic hepatic lobes without reperfusion, immunohistochemical studies were performed to detect changes in the localization of HMGB1. In sham-operated livers, HMGB1 was predominantly confined to the nuclei of hepatic parenchymal cells (Fig. 4A). However, after 60 min of warm ischemia without reperfusion, cytoplasmic HMGB1 expression was seen to be significantly increased in hepatocytes of both PBS-treated and scrambled siRNA-treated control mice (Fig. 4A). In contrast, knockdown of nuclear HMGB1 expression by pretreatment with HMGB1-siRNA markedly reduced the degree of ischemia-induced translocation of nuclear HMGB1 to the cytoplasm (Fig. 4A). Western blot analysis of hepatic cytoplasmic proteins further confirmed the HMGB1 translocation following ischemia. HMGB1-siRNA-treated livers that had undergone 60 min of ischemia had only a slightly higher amount of HMGB1 in the cytoplasm than did the sham-operated livers (Fig. 4B). In contrast, a significant amount of cytoplasmic HMGB1 was detected in both PBS-treated and scrambled siRNA-treated control animals when the livers were subjected to 60 min of warm ischemia.

### Knockdown of nuclear HMGB1 expression reduces its release after hepatic reperfusion

**Figure 5:**
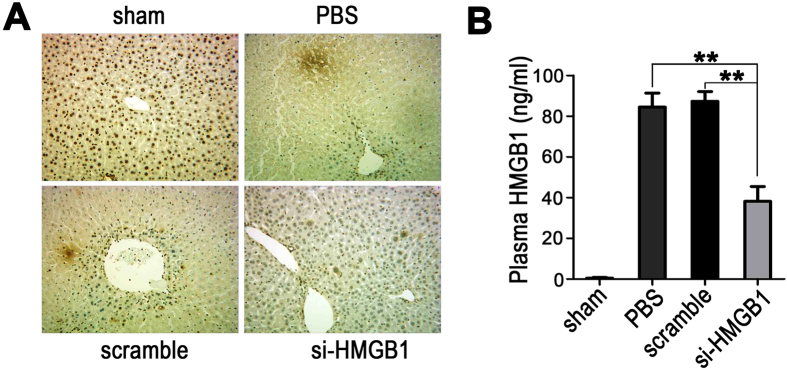
Knockdown of nuclear HMGB1 expression reduces its release after hepatic reperfusion. Mouse livers were subjected to 60 min of ischemia, followed by 6 h of reperfusion. (**A**) Immunohistochemical staining of HMGB1 from sections of livers in the unclamped mice (sham) or mice clamped and pretreated with PBS, scrambled siRNA (scramble), or HMGB1-siRNA (si-HMGB1) (original magnification, x400). Images are representative liver sections from 9 mice per group. (**B**) The plasma HMGB1 levels were measured by ELISA. The data shown are means ± SEM (**P < *0.01*; n = 6 per group).

Immunohistochemical studies were performed to detect changes in HMGB1 expression after hepatic reperfusion. After 60 min of ischemia and 6 h of reperfusion, the expression of HMGB1 in both the PBS-treated and scrambled siRNA-treated control groups was markedly attenuated, as compared to 0 h after reperfusion (Fig. 5A); this decrease may have been the result of the aggravated disruption of hepatocytes caused by reperfusion injury. In the HMGB1 siRNA-treated group, the majority of the hepatocytes remained intact and showed low levels of HMGB1 expression in both the nucleus and cytoplasm at 6 h after reperfusion (Fig. 5A), indicating that knockdown of nuclear HMGB1 expression effectively reduced the degree of nuclear-cytoplasmic translocation of HMGB1 after hepatic reperfusion.

To assess the degree of HMGB1 release into the circulation of the mice, the plasma HMGB1 levels were measured by ELISA after 6 h of hepatic reperfusion. Sham-operated animals had undetectable plasma HMGB1 levels (Fig. 5B); however, a significant amount of plasma HMGB1 was detected in both the PBS-treated and scrambled siRNA-treated control groups when the livers were subjected to 60 min of ischemia and 6 h of reperfusion. In contrast, pretreatment with HMGB1-siRNA significantly limited the increase in plasma HMGB1 values after I/R, suggesting that knockdown of nuclear HMGB1 expression can reduce HMGB1 release after hepatic reperfusion.

### Gene silencing of HMGB1 inhibits the up-regulation of HMGB1 receptors after hepatic I/R

**Figure 6:**
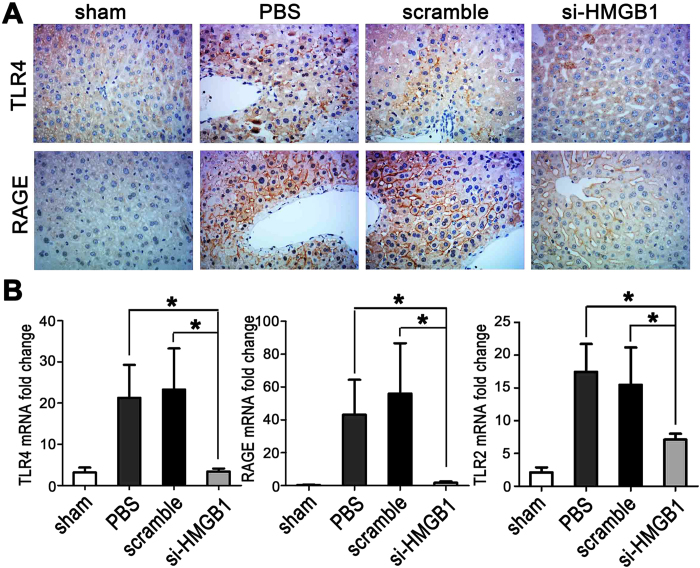
Gene silencing of HMGB1 inhibits the up-regulation of HMGB1 receptors after hepatic I/R. Mouse livers were subjected to 60 min of ischemia, followed by 6 h of reperfusion. (**A**) Immunohistochemical staining of TLR4 and RAGE from sections of livers in the unclamped mice (sham) or mice clamped and pretreated with PBS, scrambled siRNA (scramble), or HMGB1-siRNA (si-HMGB1) (original magnification, x400). Images are representative liver sections from 9 mice per group. (**B**) Real-time PCR was performed to measure mRNA expression of TLR4, RAGE, and TLR2 in the livers from the various groups (*P < *0.05*, n = 6 per group).

Since RAGE, TLR4, and TLR2 are known to be important receptors that can be activated by extracellular HMGB1 to activate proinflammatory signaling pathways^23^,^24^,^25^, we measured the expression of these HMGB1 receptors in the livers by real-time PCR and/or immunohistochemistry at 6 h after I/R. When compared to sham-operated animals, both PBS-treated and scrambled siRNA-treated control animals displayed a strong up-regulation of TLR4, RAGE, and TLR2 in response to liver I/R (Fig. 6). However, down-regulation of HMGB1 by siRNA markedly inhibited the increases in hepatic expression of these receptors when compared to control mice (Fig. 6).

### Gene silencing of HMGB1 inhibits the expression of proinflammatory cytokines and chemokines after hepatic I/R

**Figure 7:**
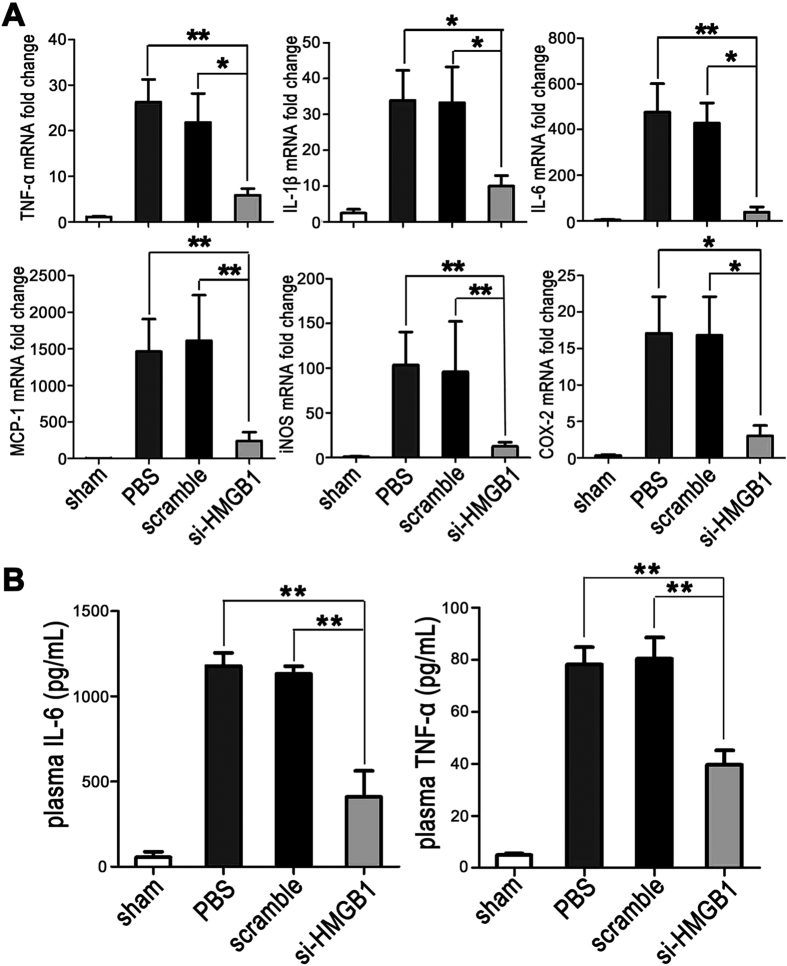
Gene silencing of HMGB1 inhibits the expression of proinflammatory cytokines and chemokines after hepatic I/R. Mouse livers were subjected to 60 min of ischemia, followed by 6 h of reperfusion. (**A**) Real-time PCR was performed to measure the mRNA expression of TNF-α, IL-1β, IL-6, MCP-1, iNOS, and COX-2 in the livers from the unclamped mice (sham) or mice clamped and pretreated with PBS, scrambled siRNA (scramble), or HMGB1-siRNA (si-HMGB1) (*P < *0.05* **P < *0.01*, n = 6 per group). (**B**) The plasma IL-6 and TNF-α levels were measured by ELISA. The data shown are means ± SEM (**P < *0.01*, n = 6 per group).

We next used real-time PCR to measure the mRNA expression of several important cytokines and chemokines downstream of HMGB1 in the livers at 6 h after reperfusion. As compared to sham-operated animals, both PBS-treated and scrambled siRNA-treated control animals showed a strong up-regulation of TNF-α, IL-1β, IL-6, MCP-1, iNOS, and COX-2 (Fig. 7A). However, animals pretreated with HMGB1-siRNA exhibited no increase or only a minimal increase in hepatic TNF-α, IL-1β, IL-6, MCP-1, iNOS, and COX-2 mRNA levels at 6 h post-I/R (Fig. 7A). In addition, the lower plasma levels of IL-6 and TNF-α, as measured by ELISA after 6 h of hepatic reperfusion, further confirmed the inhibitory effects resulting from the pretreatment with HMGB1-siRNA (Fig. 7B).

## Discussion

As a DNA-binding nuclear protein, HMGB1 can be rapidly mobilized to other sites within the cell, including the cytoplasm and mitochondria, as well as into the extracellular milieu^26^. Extracellular HMGB1 has been recognized as a critical early mediator of injury and inflammation in organ IRI^13^,^16^,^24^. Strategies that target nuclear HMGB1 may represent a more direct way to prevent nuclear-cytoplasmic translocation and release of HMGB1 and thus potentially protect against injury after I/R. However, it is surprising that hepatocyte-specific HMGB1 knockout mice have been reported to show greater hepatocellular injury after liver I/R than do control mice^19^. In contrast, unlike complete deletion, knockdown of nuclear HMGB1 expression may have no or minimal influence on its beneficial intracellular role and therefore reduce liver damage during I/R. In the present study, we used HMGB1-specific siRNA to decrease the expression of hepatic HMGB1 and have demonstrated, for the first time, that down-regulation of nuclear HMGB1 can significantly reduce the degree of nuclear-cytoplasmic translocation and release of HMGB1 during liver ischemia, and then markedly attenuate hepatic injury and inflammation after reperfusion.

siRNA provides an endogenous mechanism of cellular RNA control that is accomplished through the degradation of specific messenger RNA sequences^27^. It has been reported that intranasal delivery of HMGB1-siRNA confers target gene knockdown and robust neuroprotection in the post-ischemic brain^28^. In the present study, using the *in vivo*-jetPEI as a vector, we have clearly demonstrated by western blotting, real-time PCR, and immunohistochemical staining that our designed HMGB1-siRNA can achieve a reduction of ~60–70% in the nuclear HMGB1 expression in livers at 48 h after the treatment. The knockdown of nuclear HMGB1 expression significantly protected against hepatic IRI, as evidenced by an improvement in liver function and a marked reduction in pathological damage, including sinusoidal congestion, hepatocellular necrosis and apoptosis, and neutrophil infiltration. Huang and coworkers recently reported that mice with a selective deletion of HMGB1 from their hepatocytes show exacerbated hepatic I/R injury, with increased DNA damage and poly(ADP-ribose) polymerase (PARP) activation, indicating a role for intracellular HMGB1 in cellular protection^19^. The protective results observed in our study suggest that a certain portion of the HMGB1 remaining in the hepatocyte nuclei is capable of avoiding the increased susceptibility to cellular death following liver I/R.

Our previous studies have demonstrated that the HMGB1 that is passively released from ischemic renal parenchyma cells makes a critical contribution to the propagation of the inflammatory response and results in further renal tissue damage in a mouse renal IRI model^13^,^17^. In the present study, we first determined whether down-regulation of nuclear HMGB1 expression by siRNA could reduce the degree of nuclear-cytoplasmic translocation of HMGB1 during the period of liver ischemia. Our results demonstrated that ischemia alone can induce a significant translocation of HMGB1 from its normal site of residence in the nucleus to the cytoplasm in hepatic cells in the control groups, indicating that nuclear HMGB1 can be sensitively released by ischemic hepatocytes. In contrast, after the same stimulation by hepatic warm ischemia, knockdown of nuclear HMGB1 expression markedly reduced the translocation of HMGB1. Moreover, we also assessed the degree of HMGB1 translocation and release within the early period after hepatic reperfusion. The majority of hepatocytes remained intact and retained low levels of HMGB1 expression in both the nucleus and cytoplasm at 6 h after reperfusion in the HMGB1 siRNA-treated group, indicating that knockdown of nuclear HMGB1 expression reduces the degree of HMGB1 nucleo-cytoplasmic shuttling not only during the period of ischemia but also at critical early times after reperfusion. Also, pretreatment with HMGB1-siRNA significantly limited the increase in plasma HMGB1 values after I/R, suggesting that knockdown of nuclear HMGB1 expression also significantly reduced the release of HMGB1 to the circulation after hepatic reperfusion.

Since proinflammatory signaling pathways activated by the interaction of the released HMGB1 and its receptors are essential for mediating tissue damage after organ I/R^29^,^30^, we examined the expression of several important pattern recognition receptors and of cytokines and chemokines downstream of HMGB1 in the livers after reperfusion. Knockdown of nuclear HMGB1 expression by siRNA markedly inhibited the increases in the hepatic expression of TLR4, TLR2, RAGE, TNF-α, IL-1β, IL-6, and MCP-1 seen in control mice at 6 h after hepatic I/R. These results provided direct evidence that the attenuation of HMGB1 nuclear-cytoplasmic translocation and release is able to prevent not only the upregulation of its receptors but also the generation of these important cytokines and chemokines involved in reperfusion injury. The upregulation of TLR4, RAGE, and TLR2 in control animals is most likely a response to hepatic I/R injury. Although gene silencing of HMGB1 should not have direct influence on HMGB1 receptors, its protective effects on hepatic I/R injury may result in reduced expression of TLR4, RAGE, and TLR2 after hepatic I/R. In addition, since hepatic I/R-induced injury is associated with oxidative/nitrative stress which is indicated by the generation of iNOS and COX-2^31^,^32^, we also measured iNOS and COX-2 mRNA expression in the livers by real-time PCR. Down-regulation of HMGB1 by siRNA significantly suppressed iNOS and COX-2 mRNA expression in the livers after I/R, suggesting that the reduction in HMGB1 release may also have the capacity to inhibit oxidative/nitrative stress.

In conclusion, we have demonstrated, for the first time, that down-regulation of nuclear HMGB1 expression by specific siRNA significantly protects the liver against warm IRI by directly reducing HMGB1 release. Moreover, the use of this strategy for therapy would most likely avoid the increased susceptibility to cellular death that results from the complete deletion of nuclear HMGB1 in hepatocytes. Therefore, the use of HMGB1-siRNA may not only be helpful for better understanding the role of HMGB1 in organ IRI but may also serve to attenuate I/R-induced injury in clinical organ transplantation.

## Materials and methods

### Animals

Male BALB/c (*H*-*2*^*d*^) mice (6–8 weeks old, 20–25 g) were obtained from the Institute of Laboratory Animal Sciences, Chinese Academy of Medical Sciences (Beijing, China) and maintained under specific pathogen-free conditions. All of the experiments were performed under the guidelines of Tongji animal use regulations and approved by the institutional animal care and use committee (IACUC) at the Tongji Medical College, Huazhong University of Science and Technology.

### HMGB1-siRNA design

Targeting sequences of the mouse HMGB1 gene were selected. The oligonucleotides contained the sense and antisense target sequences, with restriction enzyme sites at the end of the strands. The sequences were: 5′-GGCUGACAAGGCUCGUUAU-3′ (sense) and 5′-AUAACGAGCCUUGUCAGCC-3′(antisense). The oligonucleotides and control siRNAs were synthesized by Guangzhou RiboBio Co., Ltd (Guangdong, China).

### In vivo silencing of the HMGB1 gene

*In vivo* gene silencing was achieved using the *in vivo*-jetPEI (Polyplus-transfection, France) as a vector^33^. In brief, 6.4 μL of *in vivo*-jetPEI and 50 μg of HMGB1-specific siRNA or scrambled siRNA were mixed and then incubated for 15 min at room temperature, and the mixture was then injected into the mice through one of the lateral tail veins.

### Hepatic IRI model

A nonlethal model of segmental (~70%) hepatic warm I/R was used as previously reported^11^. In brief, mice were anesthetized via an intraperitoneal injection of 1% sodium pentobarbital solution (6 ml/kg). Following abdominal incision, the arterial blood supply to the middle and left lobes of the liver was occluded with a microvascular clamp for 60 min. During the procedure, the mice were kept well-hydrated with warm saline and at a constant temperature of 32 °С in an infant incubator. Mice were sacrificed at 0 h or 6 h after reperfusion, blood samples were collected, and the ischemic portions of the liver tissue were harvested for further analysis. In the sham operation group, the mice underwent the same protocol without the vessel occlusion, and they were again sacrificed at 0 h or 6 h after the sham operation.

### Assessment of liver function

Blood samples were obtained from the inferior vena cava 6 h after reperfusion. Serum ALT and AST levels were measured in the core laboratory of Tongji Hospital (Wuhan, China) to assess liver function.

### Histological examination

Liver tissue samples were fixed in formalin and then embedded in paraffin. Five-micrometer sections were stained with H&E by standard methods. The severity of liver IRI was evaluated by a pathologist in a blinded fashion using Suzuki’s criteria, with the following scale: 0, no liver necrosis; 1, single-cell necrosis; 2, up to 30% lobular necrosis; 3, up to 60% lobular necrosis; 4, more than 60% lobular necrosis^34^.

### Immunohistochemical staining

Liver MPO activity was evaluated by immunohistochemical staining as described previously^22^. Neutrophil infiltration was assessed quantitatively by counting the number of neutrophils per high-powered field (x400) over 10 fields, then averaging the neutrophil counts.

Immunohistochemical staining for HMGB1 expression in liver tissue was performed using a technique that we have described previously^13^. Rabbit monoclonal antibody against HMGB1 (ab79823, Abcam, Cambridge, UK; 1:250) was used as the primary antibody, and horseradish peroxidase (HRP) polymer-conjugated goat anti-rabbit antibody (MultiSciences, China) as the secondary antibody.

### Apoptosis assays

Apoptotic cells in IRI liver tissue were detected by terminal deoxynucleotidyl transferase–mediated uridine triphosphate nick-end labeling assays using the *In Situ* Cell Apoptosis Detection Kit (Boster, China), as described previously^13^.

### Western blotting

The total cellular protein in the liver tissues was isolated using cell lysis buffer (Beyotime, China), and the cytoplasmic proteins were isolated using a Nuclear and Cytoplasmic Protein Extraction Kit (Beyotime, China) according to the manufacturer’s instructions. Protein concentrations were determined with a Bicinchoninic Acid (BCA) Protein Assay Kit (Beyotime, China). Thereafter, the proteins were stored at −80 °С for western blot analysis.

Total or cytoplasmic HMGB1 in liver tissues was determined by western blotting as described^17^. Rabbit anti-HMGB1 monoclonal antibody (ab79823, Abcam, UK; 1:5000) was used as the primary antibody, and HRP-conjugated goat anti-rabbit IgG (MultiSciences, China; 1:5000) as the secondary antibody. β-actin was used as an intrinsic quality control. The chemiluminescence was detected by an automatic exposure machine (Syngene, UK). The density of bands was quantified using Labworks image acquisition and analysis software (UVP, Upland, USA).

### Real-time PCR analyses of mRNA

To determine the expression levels of TNF-α, IL-1β, IL-6, MCP-1, iNOS, COX-2, TLR4, RAGE, TLR2, and β-actin in liver tissues, total RNA was extracted using the TRIzol reagent (Invitrogen, Shanghai, China) according to the manufacturer’s instructions; 500 ng of total RNA was used for reverse transcription with a Reversed First Strand cDNA Synthesis Kit (Thermo Scientific, Waltham, MA) to generate first-strand cDNA. The PCR reaction mixture was prepared using a Maxima SYBR Green/ROX qPCR Master Mix Kit (Thermo Scientific) with the primers shown in Table 1. The relative quantities of amplified cDNAs were analyzed by the Sequence Detection System software (PE Applied Biosystems, Foster City, CA), and the target values were normalized to β-actin mRNA.

### Enzyme-linked immunosorbent assays

Serum HMGB1 levels in the mouse were measured using a commercial ELISA kit (Westang, China). Serum TNF-α and IL-6 levels were assessed using ELISA kits (eBioscience) according to the manufacturer’s instructions.

### Statistical analysis

Data are expressed as means ± SEM. Statistical comparisons between two groups were performed using a two-tailed Student’s *t*-test; multiple groups were analyzed by one-way ANOVA analysis (GraphPad Prism 5.0; GraphPad Software; GraphPad, Bethesda, MD). Differences were considered significant at *p* < 0.05.

## Additional Information

**How to cite this article:** Zhao, G. *et al*. Down-regulation of nuclear HMGB1 reduces ischemia-induced HMGB1 translocation and release and protects against liver ischemia-reperfusion injury. *Sci. Rep.* **7**, 46272; doi: 10.1038/srep46272 (2017).

**Publisher's note:** Springer Nature remains neutral with regard to jurisdictional claims in published maps and institutional affiliations.

## Figures and Tables

**Table 1:** Primers used to amplify cDNAs for mice.

Gene	Forward(5′-3′)	Reverse(5′-3′)
TNF-α	TATGGCTCAGGGTCCAACTC	GGAAAGCCCATTTGAGTCCT
IL-1β	GCACTACAGGCTCCGAGATGAA	GTCGTTGCTTGGTTCTCCTTGT
IL-6	AGTGGCTAAGGACCAAGAC	ATAACGCACTAGGTTTGCCGA
MCP-1	CACTCACCTGCTGCTACTCATT	TGTCTGGACCCATTCCTTCTTG
iNOS	ATTCACAGCTCATCCGGTACG	GGATCTTGACCATCAGCTTGC
COX-2	GTGTATCCCCCCACAGTCAAA	ACACTCTGTTGTGCTCCCGAA
TLR-2	TCTAAAGTCGATCCGCGACAT	CTACGGGCAGTGGTGAAAACT
TLR-4	ATGCTGCAACTGATGTTCCTTC	GATGTTAGACCTTTCTTCCTCCC
RAGE	GAATCCTCCCCAATGGTTCCC	CCCTCGCCTGTTAGTTGCCCG
β-actin	CTGAGAGGGAAATCGTGCGT	CCACAGGATTCCATACCCAAGA
